# Comparative transcriptomic profiling in the pulp and peel of pitaya fruit uncovers the gene networks regulating pulp color formation

**DOI:** 10.3389/fpls.2022.968925

**Published:** 2022-08-03

**Authors:** Xiaomei Li, Yayuan Tang, Li Li, Guidong Liang, Jing Li, Chaoan Liu, Xuemei He, Jian Sun

**Affiliations:** ^1^College of Chemistry and Bioengineering, Guilin University of Technology, Guilin, China; ^2^Agro-food Science and Technology Research Institute, Guangxi Academy of Agricultural Sciences, Nanning, China; ^3^Guangxi Key Laboratory of Fruits and Vegetables Storage-processing Technology, Nanning, China; ^4^Horticultural Research Institute, Guangxi Academy of Agricultural Sciences, Nanning, China; ^5^Guangxi Academy of Agricultural Sciences, Nanning, China

**Keywords:** pitaya fruit, pulp color formation, red peel, transcriptome, gene network

## Abstract

Pitaya (genus *Hylocereus*) is a popular fruit. To develop pitaya fruit with greater marketability and high nutritional value, it is important to elucidate the roles of candidate genes and key metabolites that contribute to the coloration of the pitaya pulp and peel. By combining transcriptome and biochemical analyses, we compared and analyzed the dynamic changes in the peel and pulp of *H. undatus* (white pulp) and *H. polyrhizus* (red pulp) fruits at four key time points during ripening. Differential expression analysis and temporal analysis revealed the difference regulation in pathways of plant hormone signal transduction, phenylpropanoid biosynthesis, and betalain biosynthesis. Our results suggest that color formation of purple-red peel and pulp of pitaya is influenced by betalains. Increased tyrosine content and fluctuation in acylated betalain content may be responsible for pulp color formation, while some of the key genes in this network showed differential expression patterns during ripening between white pulp and red pulp fruits. The data and analysis results of this study provide theoretical basis for the red color formation mechanism of pitaya, which will facilitate future work to improve pitaya fruit physical appearance and marketability.

## Introduction

Pitaya (genus *Hylocereus*) originated in Latin America and is usually cultivated in tropical and subtropical habitats (Mizrahi et al., 1997; Suh et al., 2014). Pitaya species have several cultivars with different peel and pulp colors (Suh et al., 2014). *Hylocereus undatus* and *Hylocereus polyrhizus* are the most widely cultivated species. The pulp color of *H. undatus* is white, whereas that of *H. polyrhizus* is red. In addition to their exotic appearance and striking color, pitaya fruits are known for their nutritional and health benefits (Chen and He, 1998; Li et al., 2003; Kim et al., 2011). Pitaya fruit is rich in dietary fiber, minerals, proteins, unsaturated fatty acids, and multiple antioxidants, including vitamin C, organic acids, flavonoids, and betalains (Magdalena et al., 2001; Mahattanatawee et al., 2006; Hong et al., 2010; Hua et al., 2018; Wu et al., 2019).

Red pulp pitaya is more popular than white pulp pitaya because of its striking color and high nutritional value (Xi et al., 2019). In previous reports, anthocyanins and betalains were thought to influence the purple-red color of pitaya fruits. Anthocyanins are phenolic compounds that turn plants from red to purple, and they have antioxidant, anti-mutation, cardiovascular disease prevention, liver protection, tumor cell metastasis inhibition, and other health-promoting effects (Pietta, 2000; Tanaka et al., 2008; Ellinger et al., 2012; Poulose et al., 2012; Pojer et al., 2013). Betalains are nitrogen-based water-soluble pigments derived from tyrosine and are associated with the red flesh color of pitaya fruit (Gandía-Herrero and García-Carmona, 2013). In addition to producing red fruits, betalains are considered important antioxidants that protect consumers from oxidative stress and some degenerative diseases (Kanner et al., 2001). They are also thought to play a role in plant defenses against different biotic and abiotic stresses (Nakashima et al., 2011; Jain et al., 2015; Jain and Gould, 2015; Polturak et al., 2017). Existing evidence that anthocyanin and betaglycoside affect the color formation of pitaya fruit is insufficient. In particular, the pigment that determines the color of red flesh is controversial. Fan et al. (2020) suggested that anthocyanin pathways contribute to fruit flesh coloration in pitaya. However, Pucker et al. (2021) indicated that Fan et al.’s (2020) data are not credible due to the absence of the LC-MS/MS spectra and co-elution/fragmentation of the authentic standard comparison. And their reanalysis of the data sets provided by Fan et al. (2020) indicated that there are additional problems, including the failure to detect betalains in an untargeted metabolite analysis, accumulation of reported anthocyanins that independent of the fruit color, and the absence of key anthocyanin synthesis genes from quantitative real-time PCR (qPCR) data. Moreover, it is generally believed that anthocyanins and betalains cannot coexist in the same species (Pucker et al., 2021).

Metabolome and transcriptome analyses of the pulp and peel of two pitaya species have been reported (Hua et al., 2018; Zhou et al., 2020). However, previous studies of pitaya fruit using transcriptome sequencing technology have focused on comparing the gene expression profiles of pitaya variations at specific time points (Zhou et al., 2020). Fluctuations in gene expression, the role of genes during pitaya development, and the mechanism that determines the color formation of purple-red pitaya pulp and peel remain unclear. We believe that dynamic data can help us obtain more information about fruit color formation in pitaya. Here, we sequenced the transcriptome in the peel and pulp of *H. polyrhizus* and *H. undatus* pitaya fruit at four key time points during the ripening process. We then analyzed the fluctuations in gene expression during fruit ripening and found that the development of white pitaya was promoted by plant hormones for a longer period than that of red pitaya. In addition, our data provides much information for studying the color formation mechanism of pitaya fruit flesh. Combined with the results of the variations in physiochemical characteristics and biochemical composition, we suspected that betalains play important roles in determining the color of both the red peel and pulp of pitaya fruits, and fluctuations in the biosynthesis of tyrosine and acylated betalain may be important factors affecting the accumulation of betalains in pitaya.

## Materials and methods

### Plant materials

Two different types of pitaya fruits, white pitaya (*H. undatus*) and red pitaya (*H. polyrhizus*), were collected from The Guangxi Academy of Agricultural Sciences on June 20, 2020. The maturity time of pitaya is 35 days after pollination (DAP). Three biological replicates of 48 peel and pulp samples of each type were obtained at 20, 25, 30, and 35 DAP and immediately placed in liquid nitrogen until use.

### Measurement of physical and chemical characteristics

The total soluble sugar, vitamin C, amino acids, flavonoids, betalains, soluble proteins, and phenylalanine content in the extract were determined using previously published methods (Arivalagan et al., 2021). The total soluble sugar content was measured using the phenol-sulfuric acid method. Vitamin C content was determined using 2,6-dichlorophenol-indophenol (DCPIP) method. Amino acid determination was performed using the ninhydrin method. Flavonoid-level was determined using the aluminum chloride/sodium nitrite method. Betalain content was measured spectrophotometrically at 478 nm for betaxanthins and 538 nm for betacyanins. The total polyphenol and carotenoid contents were determined using a UV spectrophotometer following previously described methods (Kim et al., 2011; Fidrianny et al., 2014). The Bradford method was used to estimate soluble protein concentration. Phenylalanine levels in the extract were measured over time using a commercially available kit. All measurements were performed in triplicate as a minimum.

### RNA isolation, cDNA library preparation and RNA-Seq

Total RNA was extracted from each sample using the TRIzol reagent kit (Invitrogen, Carlsbad, CA, United States) following the manufacturer’s protocol. The concentration and quality of the total RNA were determined using a Qubit2.0 fluorometer and agarose gel electrophoresis, respectively. cDNA library products of each sample were generated in six steps: mRNA purification, first-strand cDNA synthesis, second-strand cDNA synthesis, cDNA fragment purification, end repair, adaptor ligation, and addition of index codes. The cDNA, with a length of approximately 200 bp, was sequenced on an Illumina Novaseq 6000 platform.

### Global and differential gene expression analysis

To obtain high-quality clean data, we performed quality checks and filtered the raw data to avoid non-meaning read interruptions using fastp (Version 0.18.0) (Chen et al., 2018). The parameters of FASTQ software for removing reads were reads containing adapters, the proportion of N content within reads exceeding 10%, and *Q*-value ≤ 20 bases. The obtained clean reads were then assembled *de novo* using Trinity (Grabherr et al., 2011) (Version 2.8.4) with default parameters to generate unique consensus sequences. The assembled results were validated using benchmarking universal single-copy orthologs (BUSCO). The abundance of unigene expression was then quantified and normalized to reads per kilobase per million reads. Differential expression analysis of the two samples was performed using the R package DESeq2 (Version 1.22.2). Genes with a false discovery rate (FDR) ≤ 0.05 and an absolute value of log_2_Foldchange ≥ 1 were used as the threshold to determine the significantly differentially expressed unigenes.

### Unigene annotation and function analysis

Unigenes were annotated in different databases, including NCBI non-redundant protein (Nr), Swiss-Prot protein, cluster of orthologous groups of proteins (COG/KOG), and kyoto encyclopedia of genes and genomes (KEGG) by the BLASTx (Version 2.6.0+) program. Blast2GO (Version 3.0) was used to analyze the gene ontology (GO) annotation. The differentially expressed unigenes of the pairwise comparison were mapped to GO and KEGG pathway terms and compared with unigenes with the whole genes in pitayas to identify significantly enriched GO and KEGG pathway terms based on the hypergeometric test (adjusted *P*-value < 0.05).

### Temporal analysis

Temporal analysis was done to determine the trend of gene expression patterns of multiple samples among a series of time points through a cluster approach. We performed a temporal analysis to assess the expression pattern of differential expression genes (DEGs) in two steps. The expression value of each sample was normalized to 0, log_2_ (v1/v0), and log_2_ (v2/v0). These DEGs were clustered by Short Time-series Expression Miner software (STEM) (Ernst and Bar-Joseph, 2006) with a maximum unit change in model profiles between time points of 1, maximum output profile number of 20, and minimum ratio for fold changes of DEGs of no less than 2. The clustered profiles (*p* ≤ 0.05) were used for functional annotation analysis through the hypothesis test. The GO terms or KEGG pathways with *Q*-value ≤ 0.05 was considered as significantly enriched GO terms or pathways for these significantly clustered profiles.

### Quantitative real-time PCR analysis

Quantitative real-time (qrt-PCR) was performed to determine the expression of specific genes. We extracted RNA from two types of pitaya fruit with TRIzol reagent (Invitrogen, Carlsbad, CA, United States) and converted RNA to cDNA using a cDNA synthesis kit (Thermo Scientific, United States). The cDNA sample was diluted 10 times as a qRT-PCR template. Specific primer sets of unigene sequences for qRT-PCR were designed using Primer Premier software (5.0) (Supplementary Table 1). Real-time PCR (RT-PCR) was conducted with a the CFX Connect™ Real-Time PCR detection system (Bio-Rad, United States) using Real Universal Color PreMix (SYBR Green) (TIANGEN, China). The amplification program for the PCR reaction was set based on previous studies as follows: 95°C for 3 min; 40 cycles of 95°C for 10 s and 55°C for 60 s. We used PTBP gene as an internal control of *H. polyrhizus* and DNAJ as an internal control of *H. undatus* for gene expression normalization (Zheng et al., 2020) and the 2 ^(–ΔCt)^ algorithm to estimate the expression value of unigenes.

## Results

### Variations in physiochemical characters and biochemical composition

**FIGURE 1:**
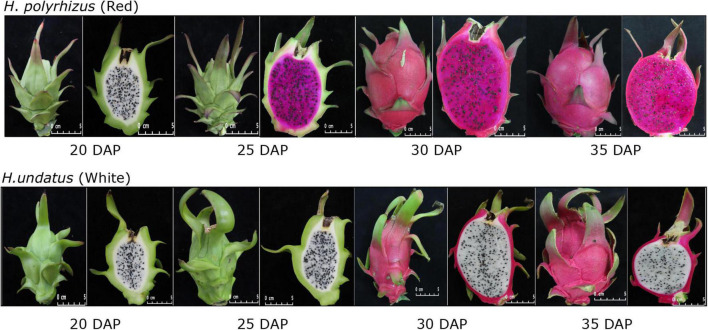
Different developmental stages of white and red pitaya.

The peel of the two commercial varieties appeared green color from 20 DAP to 25 DAP, then became to red at 30 DAP. The pulp color of *H. polyrhizus* was white at 20 DAP and change to red started at 25 DAP, while *H. undatus* presented white pulp during various growth periods (Figure 1).

**FIGURE 2:**
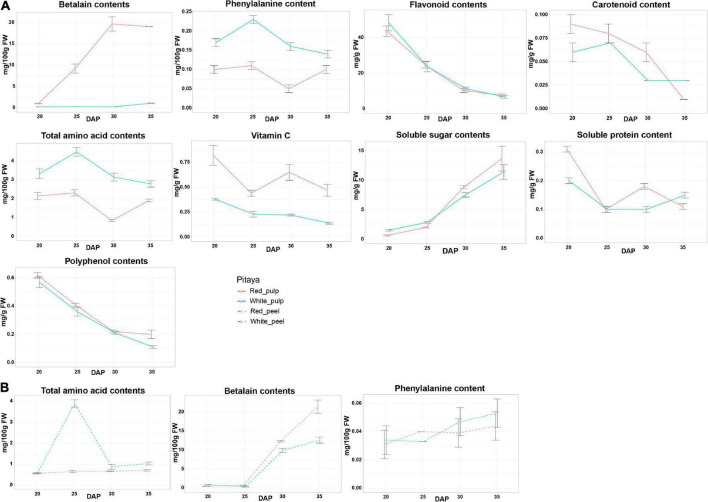
Physiochemical characters and biochemical composition in pulps **(A)** and peels **(B)** of white and red pitaya.

The patterns of each biochemical component are shown in Figure 2. In fruit flesh, the levels of flavonoids and polyphenols gradually decreased, whereas the soluble sugar content increased during fruit ripening. In red pulp, betalain peaked during fruit ripening but slightly decreased during over-ripening. Interestingly, the betalain content in the white pulp remained low from 20 to 35 DAP. In addition, the vitamin C content of the red pulp was markedly higher than that of the white pulp. From 20 to 35 DAP, the phenylalanine and amino acid contents in the white pulp were higher than those in the red pulp.

In fruit peel, the total amino acid content peaked at 25 DAP in white peel and was rapidly restored to the baseline value from 30 DAP, while the abundance of total amino acid content in red peel was below 1 mg/100 g FW during growth periods (Figure 2B). Unlike the change trend of the betalain content detected in fruit flesh, the betalain content in the peel was unchanged from 20 to 25 DAP, after that stage the value gradually increased, and the betalain content in the red peel was higher than that in the white peel from 25 to 35 DAP. Phenylalanine content in the peel gradually increased during fruit ripening. These results indicate that the quality of white and red pitaya is markedly different during fruit development.

### Transcriptome sequencing and assembly

To obtain the gene expression profile during fruit ripening, we performed RNA-seq analysis for three biological replicate tissue samples of peel and pulp of two types of pitaya at four time points. Few reads defined as low-quality (around 1%) were removed, while the majority of reads of raw data were kept as clean data based on the removal threshold. The range of total raw and clean reads in each sample is from 39,939,916 to 105,302,806 and 39,267,962 to 102,787,458 (Supplementary Table 2). We identify 80,963 and 81,048 unigenes with N50 lengths of 2,039 and 1,813 bp using the Trinity software (Grabherr et al., 2011) to, respectively (Table 1). More than 60% unigenes in white and red fruits reported by BUSCO were considered complete, indicating that unigenes are of good quality for downstream analyses. We obtained 32,249 of 81,048 unigenes in red fruit were annotated by the Nr, KOG, Swiss-Prot, and KEGG databases and 31,935 of 80,963 annotated unigenes for white pitaya fruit.

**TABLE 1:** Features of unigene assemblies in two commercial *Hylocereus* varieties.

Type	Genes number	GC percentage	N50 number	N50 length	Max length	Min length	Average length	Total assembled bases
White	80,963	41.1084	11,907	2,039	34,298	201	1,029	83,349,627
Red	81,048	41.6169	12,028	1,813	25,408	201	928	75,279,808

### Global analysis of RNA-seq data

**FIGURE 3:**
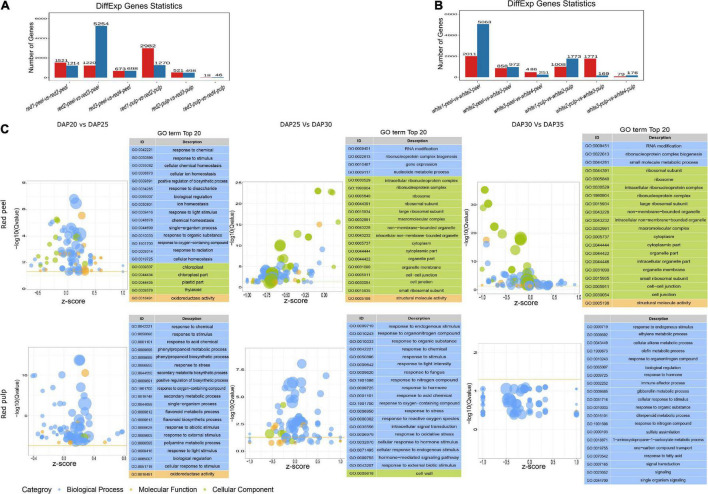
Differential expressed genes and GO term analysis. **(A)** The number of DEGs of red pitaya peel and pulp among fruit development. **(B)** The number of DEGs of white pitaya peel and pulp among fruit development. **(C)** GO terms of DEGs produced by red peel and pulp across fruit growth.

To understand the gene expression perturbation during fruit development, we compared the FPKM of each unigene between time points to identify differentially expressed genes (Figures 3A,B). The number of DEGs in peel samples extracted from red fruit gradually decreased within each interval, as well as in the pulp of white fruit. The largest change in gene expression profiles in white fruit occurred in white1-peel-vs-white2-peel, while the majority of DEG sin red fruit were identified in red2-peel-vs-red3-peel. We also observed that time 3 vs. time 4 in either the peel or pulp of the two fruit types generated fewer DEGs than in the other groups. Interestingly, the number of DEG sin the peel produced by 3rd interval comparison was higher than that in the pulp. These results indicate that the development stage of the peel might vary depending on the classification of pitaya, and the pulp of two commercial pitayas after time 3 tended to maintain a certain level of gene expression.

We performed functional annotation analysis for DEGs based on the GO and KEGG databases using the hypergeometric test algorithm. GO terms were grouped into three categories: biological processes, molecular functions, and cellular components. GO terms related to biological processes were significantly enriched by the DEGs of the red pulp across the three comparisons. Most of the DEGs produced by red peel at DAP25 vs. DAP30 and DAP30 vs. DAP35were grouped into cellular components (Figure 3C). Functional annotations of white pulp and peel DEGs were mainly related to biological processes, except that the top 20 significant functions of DEGs obtained from white peel and pulp at DAP25 vs. DAP30 were changed based on fruit development (Supplementary Figure 1). The specific top 20 GO terms for each comparison are shown in Supplementary Figure 2. Although the number of DEGs differed between the two types of pitaya fruit, most of these genes participated in the biological process of pitaya development, according to GO term enrichment analysis.

**FIGURE 4:**
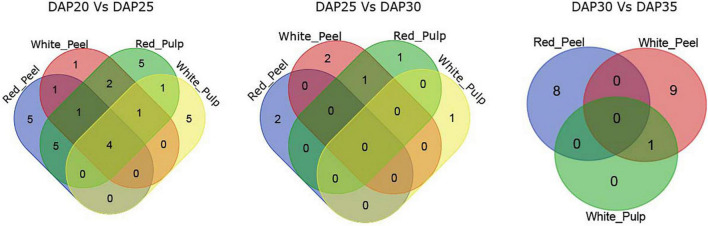
Overlapping enriched kyoto encyclopedia of genes and genomes (KEGG) pathways among pitaya development of DEGs between white and red pitaya.

The majority of DEGs among the two fruit growth stages were correlated with metabolism based on KEGG pathway enrichment analysis (Supplementary Figures 3, 4). We observed four common significant pathways identified by DEGs in fruit tissue samples at DAP20 and DAP25: plant hormone signal transduction, biosynthesis of secondary metabolites, metabolic pathways, and phenylpropanoid biosynthesis (Figure 4). The number of pathways in the DAP25 vs. DAP30 and DAP30 vs. DAP35 groups was less than that in the DAP20 vs. DAP25 group, and only one overlapping pathway appeared in the white peel and red pulp, white pulp, and white peel, respectively. These results suggest that the various features of different tissue types may depend on these unique pathways.

### Temporal analysis of differential expression genes

A previous study compared the gene expression profiles of pitaya variations at specific time points and neglected the role of genes during pitaya development (Zhou et al., 2020). We used temporal analysis to assess the DEG expression pattern of four groups, including 8,445, 4,998, 8,387, and 4,666 DEGs for red peel, red pulp, white peel, and white pulp, respectively, based on four time points. DEGs were clustered into 20 background clustered profiles for each group, and significant profiles were selected based on *p* ≤ 0.05. Of the 20 clustered profiles, seven were separately identified as significant clustered profiles in white pulp and peel, four in red peel and six in red pulp (Supplementary Figure 5).

**FIGURE 5:**
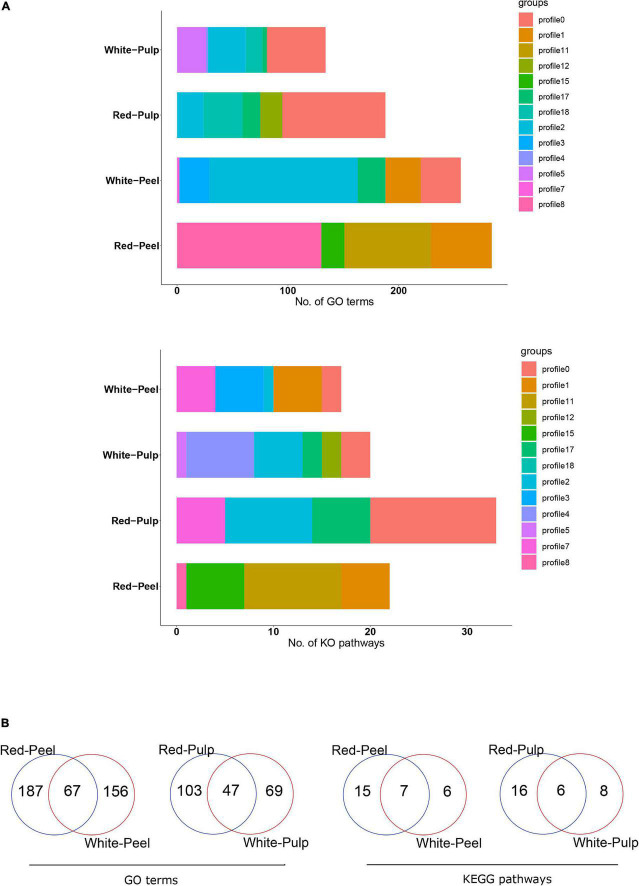
Functional annotation of significant clustered-profiles. The number of enriched categories **(A)** and their overlap of enriched categories **(B)** in significant clustered-profiles are presented.

We further performed a functional enrichment analysis to understand the biological pathways of these significant profiles. Several biological pathways were replicated within the various profiles. Genes involved in profile8 of red-peel and profile2 of white-peel significantly participated in a larger number of GO terms, while profile11 and profile0 generated by red-peel and red-pulp included the majority of biological pathways (Figure 5A). Sixty-seven of 410 common GO terms for peel and 47 of 219 common GO terms for pulp were found by comparing the peel and pulp of the two types of pitaya (Figure 5B). Several signal pathways overlapped across the four types of tissue samples, including plant hormone signal transduction and phenylpropanoid biosynthesis (Supplementary Table 3).

### Temporal gene expression trends in key pathways during fruit ripening

**FIGURE 6:**
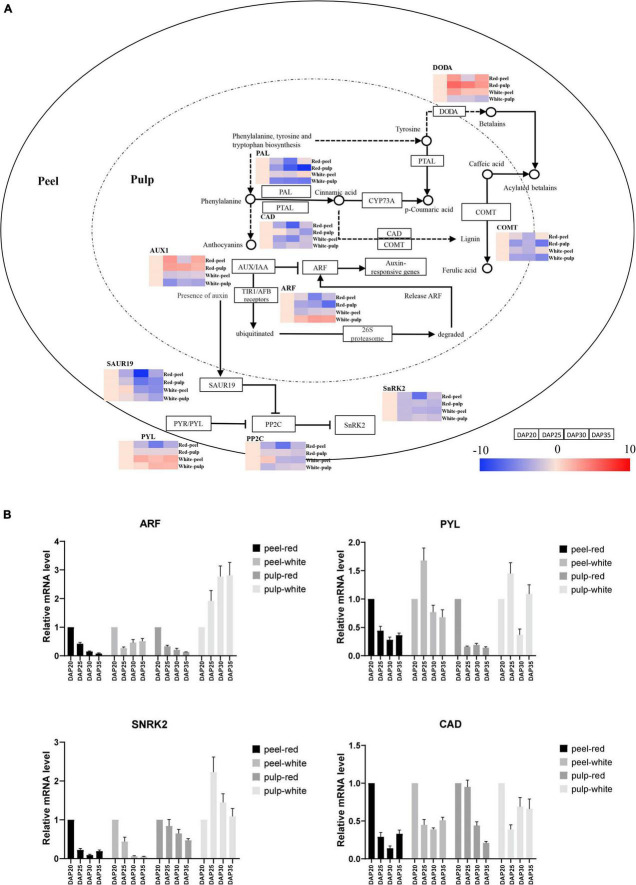
Temporal gene expression trends in key pathways during fruit ripening. **(A)** Gene expression alteration trends in plant hormone signal transduction, phenylpropanoid biosynthesis and betalain biosynthesis pathway. **(B)** Gene expression profiles validated by quantitative real-time (qrt-PCR).

To further understand the temporal gene expression trends in fruit peel and pulp during pitaya fruit development, we extracted KEGG pathway-related DEGs based on significant profile functional analysis. Figure 6A illustrates the series of expression changes of DEGs in the plant hormone signal transduction, phenylpropanoid biosynthetic and betalain pathways. Heatmaps are shown to represent the expression trends of DEGs involved in the significant profiles. The plant hormone signal transduction pathway processes the products of eight signaling pathways: tryptophan metabolism, Zeatin biosynthesis, Diterpenoid biosynthesis, Carotenoid biosynthesis, Cysteine and methionine metabolism, Brassinosteroid biosynthesis, α-Linolenic acid metabolism, and Phenylalanine metabolism. Auxin regulates cell division, expansion, and differentiation to influence all aspects of plant growth and development (Wang and Estelle, 2014). The expression of AUX1 at each time point was upregulated in red pulp and red peel excepting downregulation at DAP30 in red peel. And expression of AUX1 in white pulp and white peel was downregulated among time points. One auxin response factor (ARF) was upregulated in white pulp compared to that in red pulp from DAP20 to DAP35. The lowest expression level of SAUR family protein (SAUR), a crucial effector product of hormonal and environmental signals (Ren and Gray, 2015), was observed at DAP30 in the red pulp, while lowest expression was observed in the white pulp at DAP35.

The abscisic acid receptor PYR/PYL family (PYL), protein phosphatase 2C (PP2C), and serine/threonine-protein kinase SRK2 (PYL-PP2C-SnRK2) gene family are the main components of abscisic acid (ABA) signaling, which participates in the response to abiotic stressors and regulates plant growth and development (Xu et al., 2020). PYL was upregulated in white peel at DAP25, whereas it was downregulated in red peel at the same time point. The expression of PP2Cs in the red peel were downregulated from DAP 25 to DAP35, while that in the white peel was upregulated at DAP25 and downregulated at DAP30 and DAP35. The gene encoding PP2Cs in red pulp were upregulated from DAP20 to DAP30, decreased at DAP35. The DAP25 was the lowest expression time point of PP2Cs in the white pulp. The lowest expression of SnRK2 was observed in DAP30. The SnRK2 genes showed similar expression trends in red and white pulp during fruit growth, which were downregulated at DAP25 and then upregulated at DAP30 and downregulated at DAP35.

Phenylpropanoids, including lignin, suberin, flavonoids, and benzenoids, are generated from phenylalanine via the phenylpropanoid biosynthetic pathway. These compounds participate in all layers of plant responses to biotic and abiotic stimuli, which are indicators of plant stress responses depending on the variation in light or mineral treatment and can be mediators of plant resistance to pests (Vogt, 2010). We obtained expression trend of cinnamyl-alcohol dehydrogenase (CAD), whose lowest expression point was at DAP30 in red peel and white peel. The change trend of CAD in red pulp was going down from DAP20 to DAP35, while one was slightly changed after DAP25 in white pulp. Gene expressing caffeic acid 3-O-methyltransferase (COMT) were downregulated from DAP25 to DAP30 and then upregulated from DAP30 to DAP35 in both peel tissues. The lowest expression levels of COMT in both pitaya pulp samples were found at DAP35. Unlike the trend for PAL in white and red peel, PAL in white and red pulp remained downregulated at all time points.

To determine the expression trend of key genes involved in color formation, we explored the betalain pathways. We observed that the betalain biosynthesis pathway was significantly identified in red pulp based on profile KEGG annotation analysis. The L-DOPA 4,5-dioxygenase (DODA) genes of red pulp involved in the betalain pathway were upregulated from DAP20 to DAP35 and that in white pulp were downregulated, depending on the trend values (Supplementary Table 4). The mRNA level of key genes including ARF, PYL, SnRK2, CAD was further detected by qRT-PCR to validated their expression trends, which consistent with the expression trend found by RNA-seq approach (Figure 6B). These results illustrate that gene expression values change dynamically depending on the plant tissue and development stage.

## Discussion

In the present study, the biochemical characteristics and transcriptome of *H. undatus* and *H. polyrhizus* during fruit ripening were compared. In both *Hylocereus* cultivars, a significant change in gene expression profile was observed in the peel. However, the change occurred earlier in white fruit than in red fruit. There were differences in the functional annotation of genes that were significantly clustered between the two cultivars. The difference between the GO terms and KEGG pathways of the genes involved in white pitaya and red pitaya indicated significant physiological differences between the two cultivars of pitaya.

Auxin/Indole-3-Acetic acid (Aux/IAA) family, ARF family, and small auxin upregulated RNA (SAUR) are auxin early response genes (Abel et al., 1995; Guilfoyle and Hagen, 2007; Goldental-Cohen et al., 2017). Genes of the Aux/IAA and SAUR families are rapidly and transiently induced in response to auxin (Hagen and Guilfoyle, 2002). Aux/IAA family members have been shown to play a crucial role in inhibiting the expression levels of genes activated by ARFs (Abel et al., 1994; Dreher et al., 2006). Auxin-mediated transcriptional regulation has been demonstrated to be exclusively dependent on Aux/IAA function (Lavy and Estelle, 2016). In the absence of auxin, Aux/IAA proteins have been suggested to bind to ARFs and inhibit the activation of auxin-responsive genes, whereas these proteins can be ubiquitinated by interacting with TIR1/AFB receptors and subsequently degraded via the26S proteasome at high auxin levels (Weijers et al., 2005; Tan et al., 2007; Parry et al., 2009). The expression of auxin-responsive genes is regulated by the released ARFs (Weijers et al., 2005). Auxin may upregulate the expression of SAUR19 to inhibit the activity of PP2C-D phosphatases through transcription, resulting in the activation of plasma membrane H + -ATPases to promote cell expansion in *Arabidopsis* (Spartz et al., 2014). In our results, AUX1 was downregulated at DAP25 and then upregulated slightly, and ARF was continuously upregulated in white pulp, while the trends of AUX1 and ARF in red pulp were opposite to those in white pulp, suggesting that there may be a relatively large amount of ARF available to activate downstream auxin-responsive genes in white pulp. In addition, the lowest expression of SAUR appeared later in the white pulp than in the red pulp, which also indicated a difference in auxin hormone fluctuation between the two pulp types. These results suggest that white pulp is more affected by auxin and lasts longer than red pulp during pitaya ripening.

PYR/PYL receptors inhibit PP2Cs by direct binding in the presence of ABA (Ma et al., 2009; Park et al., 2009; Tischer et al., 2017). PP2C inhibition induces the activation of some SnRK2s. There are 10 members of SnRK2s in *Arabidopsis*, three of which are activated by ABA. In the absence of ABA, these SnRK2 kinases are inhibited by PP2C-dependent dephosphorylation (Umezawa et al., 2009; Vlad et al., 2009). From DAP20 to DAP25, the trends of PYL and PP2C were opposite in both red and white peels, indicating that the growth of white peel was promoted and that of red peel was promoted by hormones at this time point. It is worth noting that PP2C was significantly downregulated earlier in white pulp than in red pulp, suggesting that the growth of red pulp was inhibited earlier under the influence of hormones. These results were consistent with the fluctuations in auxin-responsive genes in the pulp.

The amino acid phenylalanine (Phe) is an example of the interconnection between primary and secondary metabolism because it can be a protein building block or a precursor of some secondary metabolites such as lignin, suberin, flavonoids, and benzenoids, which are essential for plant growth, development, and defense (Vogt, 2010; Pascual et al., 2016; Canton et al., 2020). Lignin biosynthesis is likely to influence cell wall properties; indeed, phenolics can form crosslinks between cell wall components (Abdel-Massih et al., 2007; Seymour et al., 2008). CAD and COMT are involved in lignin monomer biosynthesis (Trabucco et al., 2013). CAD is considered a highly specific marker for lignification. It can catalyze the synthesis of cinnamyl alcohols, the immediate precursors of lignins, from the corresponding cinnamaldehydes (Feuillet et al., 1995). There was no significant difference in the fluctuation trend of Phe or CAD between red peel and white peel. However, the content of Phe in white pulp was lower than that in red pulp. And compare with red pulp, CAD was downregulated earlier in white pulp. These results show the differences in the degree of lignification between white and red pulp. Therefore, we speculate that there may be differences in cell wall strength and fruit hardness between the two pitayas.

In addition, phenylpropanoid biosynthesis is important for the biosynthesis of pigments. Anthocyanins are derived from Phe, whereas betalains are tyrosine-derived pigments (Tanaka et al., 2008; Timoneda et al., 2019). *In vivo*, Tyr can be produced by the hydroxylation of phenylalanine. Notably, the expression of PAL was downregulated in red pulp from DAP20 to DAP35, while PAL expression was downregulated from DAP20 to DAP25 and then stabilized from DAP25 to DAP35 in white pulp. These results suggest that there may be more tyrosine available in the red pulp than in the white pulp. We hypothesized that this provides more precursors for the synthesis of betalains and fewer precursor of anthocyanins. The transcriptomic and metabolic analyses by Zhou et al. (2020) also suggested that the main strategy for obtaining the red color of pitaya is to increase the tyrosine content for downstream steps in the betalain pathway. In addition, it is interesting to note that caffeic acid can be linked to the glycosylation of betalains, making them acylated, similar to anthocyanins, but with a more complex structure (Heuer et al., 1994; Gao and Liu, 2006). Significantly fluctuating levels of COMT catalyze the formation of ferulic acid from caffeic acid. This fluctuation may cause a change in the caffeic acid content. Although little is known about the function of acylated betaine, we suspected that caffeic acid content may affect the content of acylated betaine, which may also be related to fruit color formation.

Notably, the betalain content in the peel of both cultivars increased during the critical period from DAP25 to DAP35 when pitaya turned from green to red, indicating the important role of betalain in determining peel color. In addition, the content of betalain in red pulp increased significantly during ripening, but no significant change was observed in white pulp, indicating betalains also plays an important role in the formation of red pulp. Pitayas contain two betalain pigments, namely red-violet betacyanins and yellow-orange betaxanthins (Cejudo-Bastante et al., 2016). Both red-violet betacyanins and yellow-orange betaxanthins are biosynthesized from tyrosine by enzymatic and spontaneous chemical reactions (Strack et al., 2003; Wybraniec et al., 2009). In previous reports, the color of the peel and pulp is generally thought to be mainly due to pigment betalains and other secondary metabolites, such as anthocyanins and carotenoids (Tel-Zur et al., 2011; Zhou et al., 2020). However, the color formation mechanism of red pulp pitaya remains unclear. Fan et al. (2020) reported the contribution of anthocyanin pathways to flesh coloration in pitaya. However, Pucker et al. (2021) pointed out a series of problems in Fan’s report, including the lack of qPCR data for key anthocyanin synthesis genes in red pitaya fruit. Furthermore, the key gene DODA of betalain biosynthesis was upregulated in red pulp, and downregulated in white pulp, indicating that the betalain biosynthesis pathway was enhanced in red pulp. Xi et al. (2019) have reported that all structural genes related to betalain biosynthesis had a higher expression in red pulp than white pulp. Here, our data provide data support for the contribution of betalain pathways to flesh and peel coloration in pitaya from both of metabolic and molecular fluctuations.

Although there were significant differences in the fluctuation of many auxin or ABA response genes between the two pitaya species, the effect of auxin and ABA on betalain accumulation has not been reported. It’s worth noting that in addition to phenylpropanoid biosynthetic pathway, shikimate pathway also provides Tyr for betalain biosynthesis (Tohge et al., 2013). Shikimate pathway can also provide L-tryptophan as the precursor of auxin synthesis (Wang et al., 2019). However, the effects of auxin and ABA on betalain biosynthesis, and how these hormone affect betalain biosynthesis remain to be further studied.

In conclusion, the differences in gene expression between the peel and pulp of *H. undatus* and *H. polyrhizus* were characterized. The development and ripening processes of the two species are regulated by the cooperation of auxin and ABA, but they are affected by different degrees and times, which may be important factors leading to their differences. The mechanism by which hormones affect pigment formation requires further investigation. Although the main mechanism of red pulp and peel color formation has not been determined, we found that betalains contribute to the color formation of red pulp and peel of pitayas, and increased tyrosine content and acylated betalain content may also be responsible for the purple-red color formation in pulp of *H. polyrhizus*.

## Data availability statement

The data presented in this study are deposited in the NCBI repository, accession number: PRJNA806509.

## Author contributions

XH, YT, and JS: writing – original draft preparation. JS: writing – review and editing. XL, LL, and GL: methodology. JL: resources. CL: the raw material providing and validation. XH and YT: data analysis. All authors contributed to the article and approved the submitted version.

## Supplementary material

Supplementary Figure 1 Top 20 significant functions of DE genes obtained from white peel and pulp based on fruit development.

Supplementary Figure 2 The specific top 20 GO terms for each comparison.

Supplementary Figure 3 KEGG pathway enrichment analysis of DE genes of red fruits during growth.

Supplementary Figure 4 KEGG pathway enrichment analysis of DE genes of white fruits during growth.

Supplementary Figure 5 Significant clustered profiles in peel and pulp of white and red fruits.
