# Supplementary figures and images for: Comparative transcriptomic profiling in the pulp and peel of pitaya fruit uncovers the gene networks regulating pulp color formation
Source: Front Plant Sci. 2022 Aug 3;13:968925. doi: 10.3389/fpls.2022.968925 (PMC9382024; doi:10.3389/fpls.2022.968925)

ARF

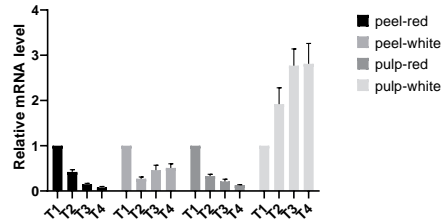

PYL

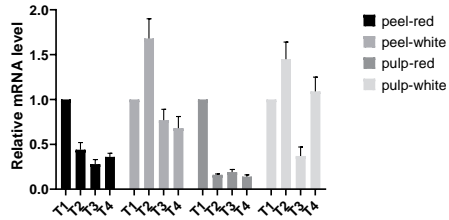

SNRK2

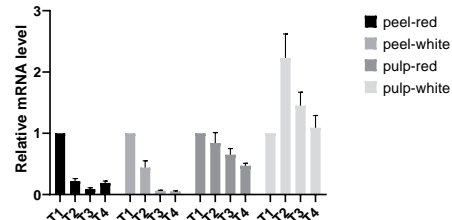

CAD

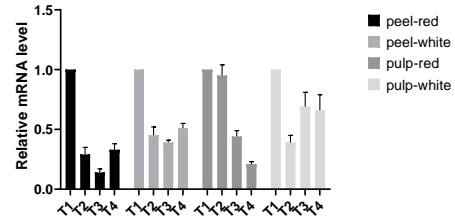

REF1

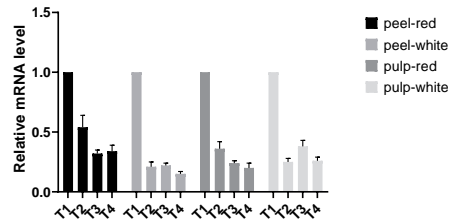

BZ1

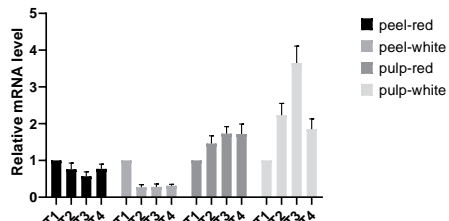

UGT79B1

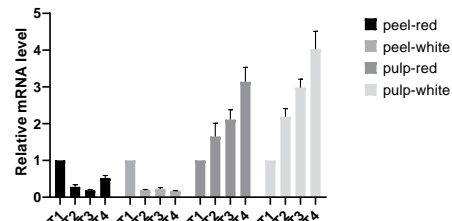

Supplement: Supplementary file 1 [file Data_Sheet_6.PDF]

DAP20 Vs DAP25

White Peel

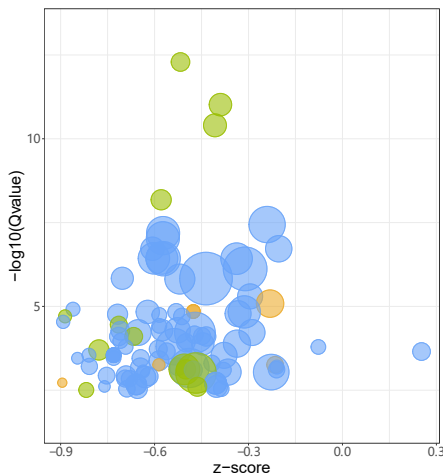

DAP25 Vs DAP30

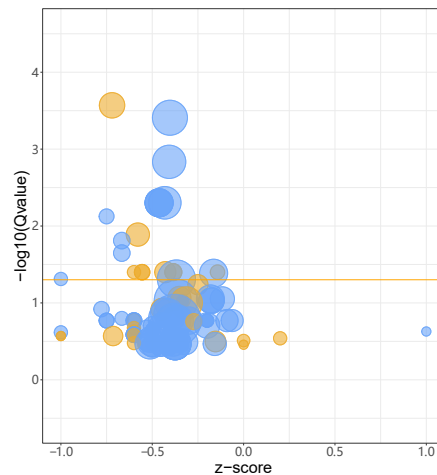

DAP30 Vs DAP35

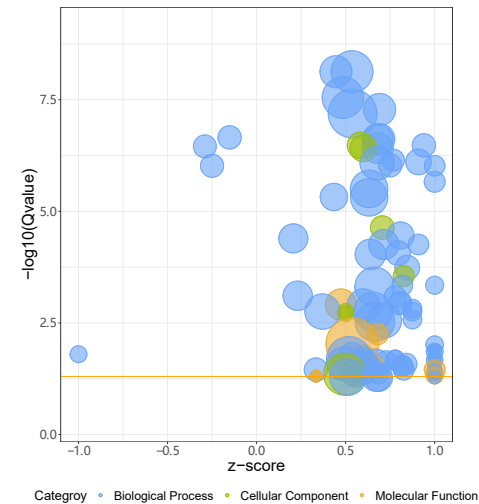

White pulp

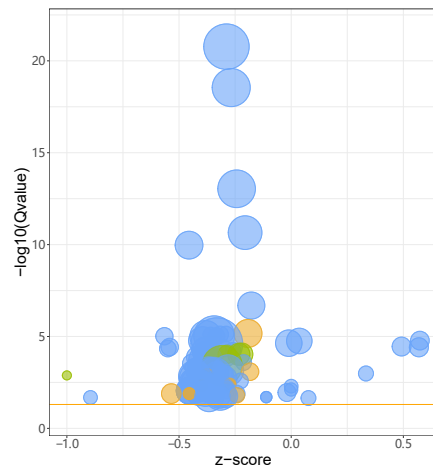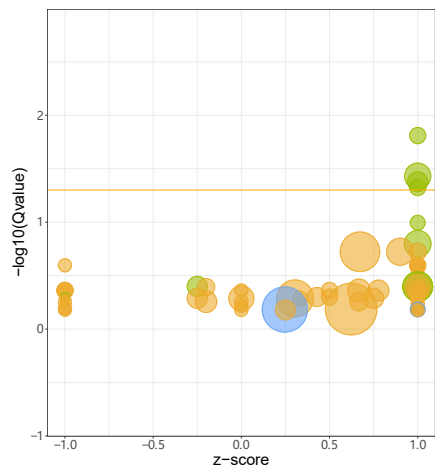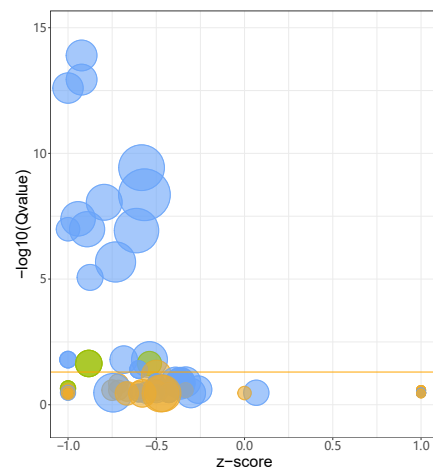

Supplement: Supplementary Figure 1 — Top 20 significant functions of DE genes obtained from white peel and pulp based on fruit development. [file Data_Sheet_1.PDF]

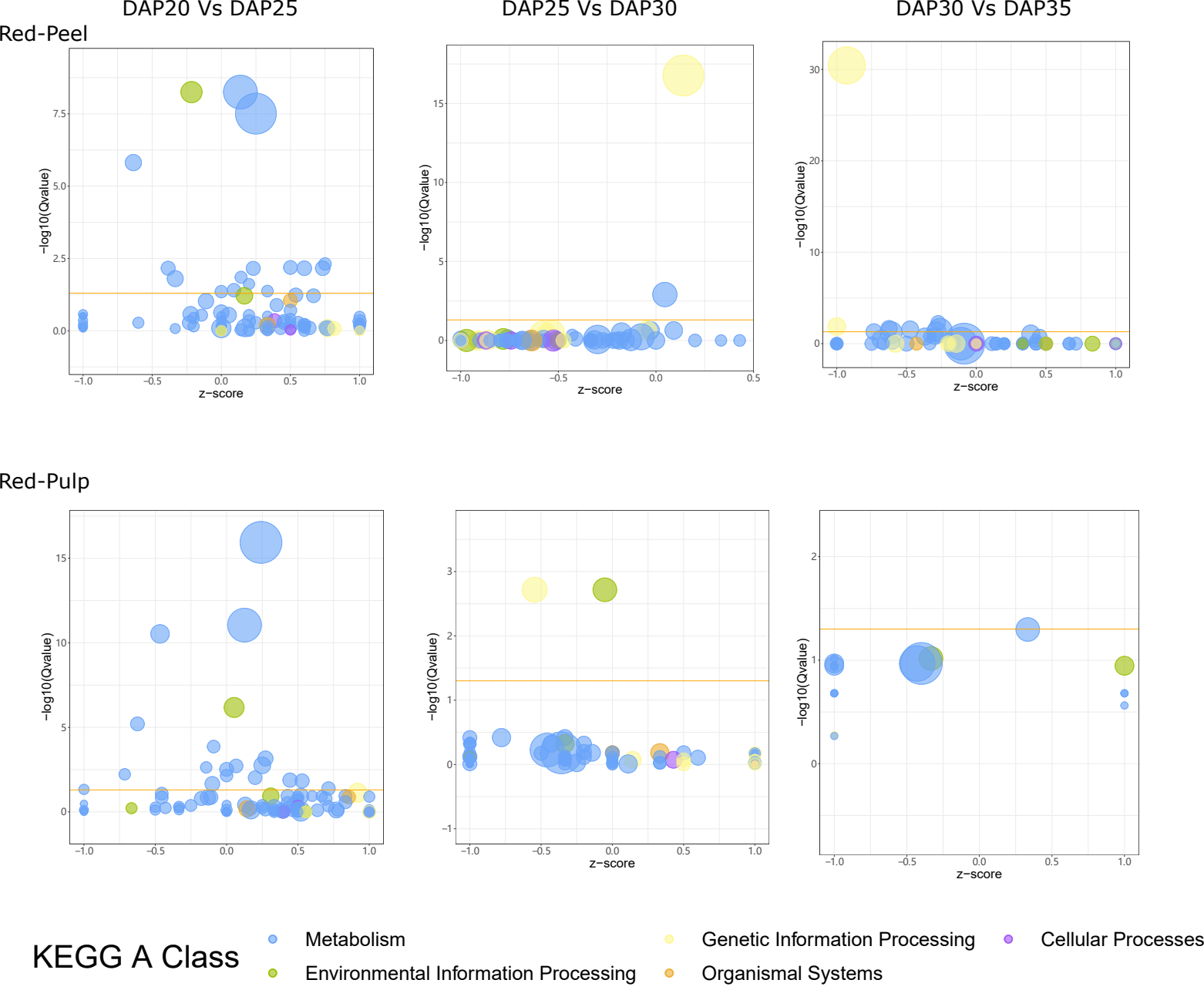

Supplement: Supplementary Figure 3 — KEGG pathway enrichment analysis of DE genes of red fruits during growth. [file Data_Sheet_3.PDF]

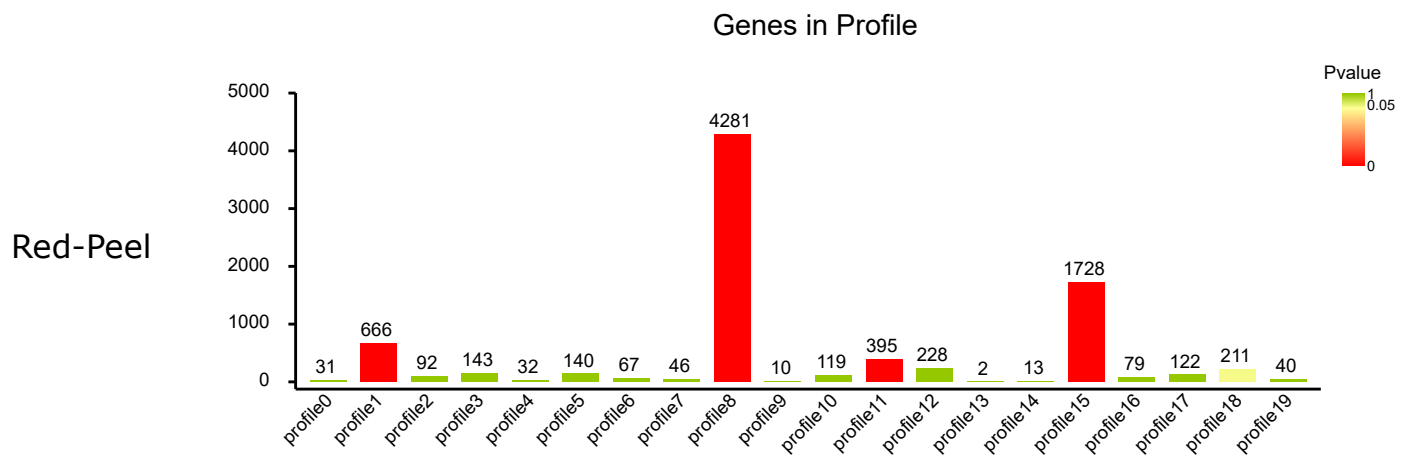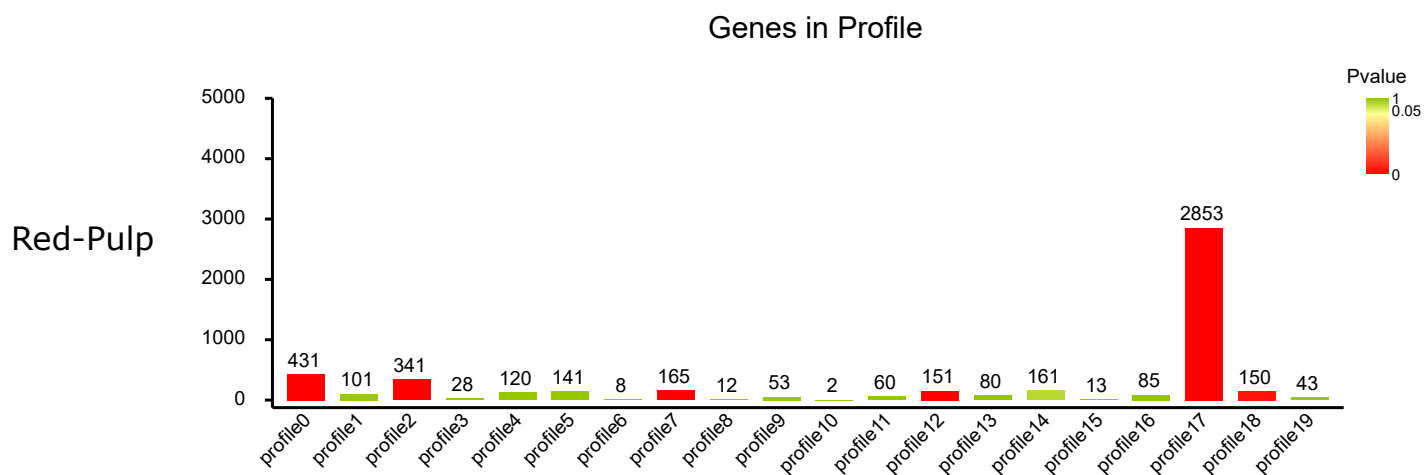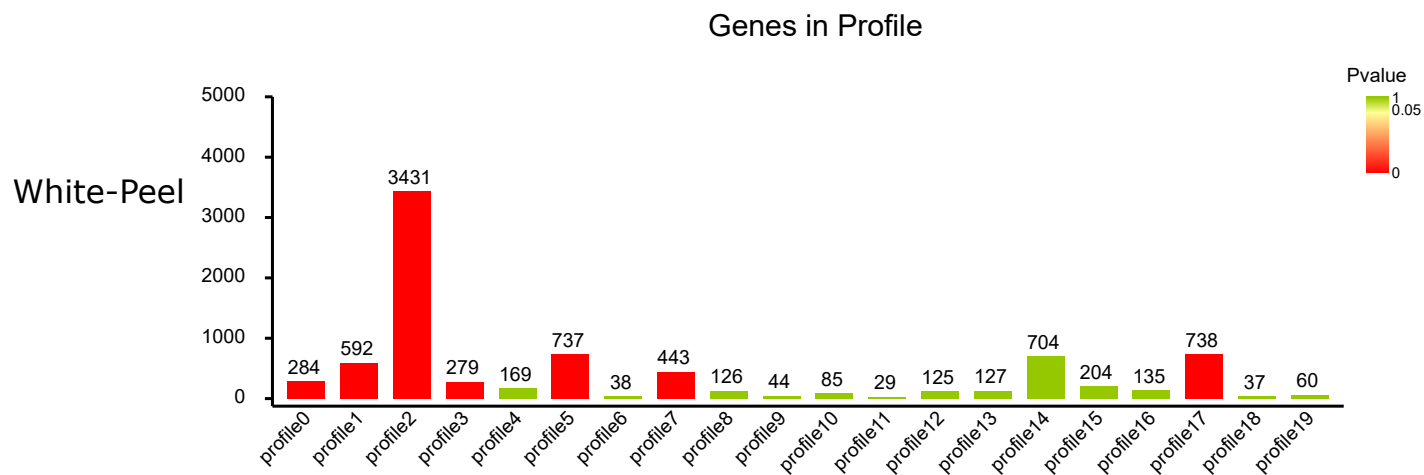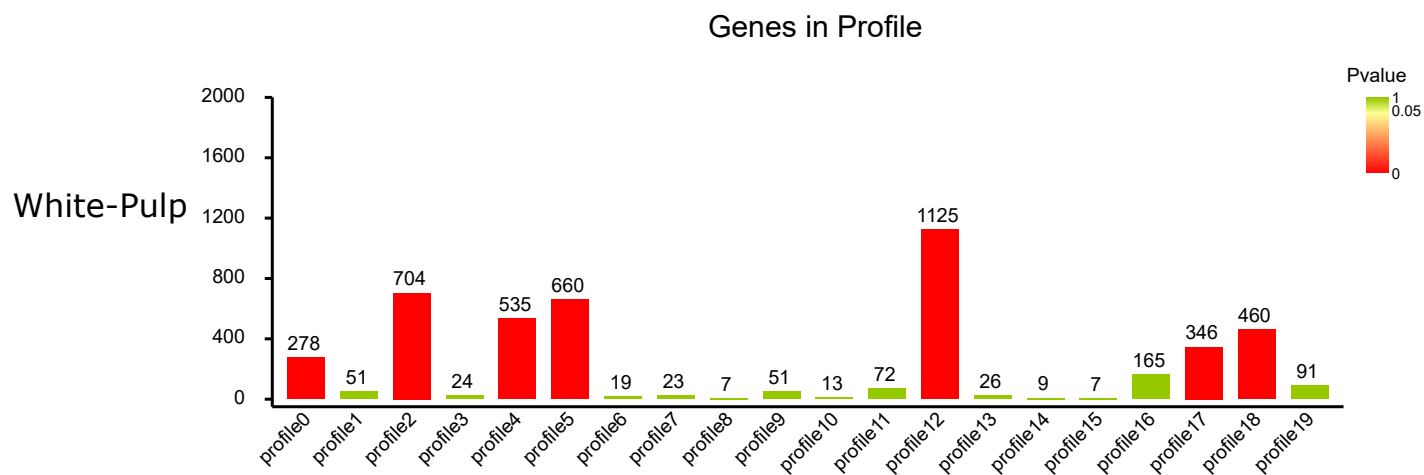

Supplement: Supplementary Figure 5 — Significant clustered profiles in peel and pulp of white and red fruits. [file Data_Sheet_5.PDF]
