# Supplementary material for: Comparative transcriptomic profiling in the pulp and peel of pitaya fruit uncovers the gene networks regulating pulp color formation
Source: Front Plant Sci. 2022 Aug 3;13:968925. doi: 10.3389/fpls.2022.968925 (PMC9382024; doi:10.3389/fpls.2022.968925)
Supplement: Supplementary file 2 [file Data_Sheet_7.docx]

**Table S1.** List of primers used for qRT-PCR analysis.

Red pitaya

| Gene | ID | Forward primer | Reverse primer |
| --- | --- | --- | --- |
| ARF | Unigene0034657 | CACCACCACAAATGGCCATCAATTA | TAATTGATGGCCATTTGTGGTGGTG |
| PYL | Unigene0036711 | CGATTGTCACAGTTCATCCAGA | CTCCTCTTTCGTGTTTCCTTCA |
| SNRK2 | Unigene0074558 | AGTCGGCAGTGCTACATTCG | AGTGACCCCACAAGACCAAAC |
| CAD | Unigene0062653 | GAGGCAGTGACATTGGAGGAA | CATCTGACTTAGCAAGCCGTTC |
| REF1 | Unigene0046018 | TGTAGTAACCGTTCTTCCCATGT | GACTGTCGGAGACCCTTTTGA |
| BZ1 | Unigene0080436 | ATGGCACCCCAATATCCTGT | GAGATCCACCTCTTCTTGAACG |
| UGT79B1 | Unigene0046218 | CCAGTTCAGGCAAAACAGGA | CGGATTACAAGGGCAATACG |
| PTBP | Unigene0076194 | AGCGTCAAATTCCAAAGCCA | ATCCAAGCCCACACCTAACT |

White pitaya

| Gene | ID | Forward primer | Reverse primer |
| --- | --- | --- | --- |
| ARF | Unigene0072171 | CCCCAGTTCTCCGTTCTCAC | GGCACTTCAAGCACATTTTCA |
| PYL | Unigene0078375 | GTTCGCATTTCGGGCTGTAG | GGTTATGGTTCGCCTCTTGTT |
| SNRK2 | Unigene0062853 | CTAAGTCAACAGTCGGGACGC | GCAACGAAGATACAAGAAAGAAGG |
| CAD | Unigene0013049 | CTGCACATAGCAGTGGAGCC | TGACAAAGTTCACGGTAGGAGATA |
| REF1 | Unigene0055442 | AACAACCCAAGCAAGCATTCA | TCCCTACGGTGGCTACAAACT |
| BZ1 | Unigene0068938 | TCCGAGTAAGATGATAGACCAACC | TGATGAGGACGCATTAGAGCC |
| UGT79B1 | Unigene0002645 | CCAGTTCAGGCAAAACAGGA | CGGATTACAAGGGCAATACG |
| DNAJ | Unigene0054867 | TACTTGCCCTTCTCAGAGCC | CTGCGATCAAAGTCCAGTGA |

**Table S2.** The status of raw reads in all sequenced samples.

| Sample | RawDatas | CleanData(%) | Adapter(%) | LowQuality(%) | polyA(%) | N(%) |
| --- | --- | --- | --- | --- | --- | --- |
| red1-peel-1 | 57500482 | 55688558 (96.85%) | 266034 (0.46%) | 1530404 (2.66%) | 0 (0.00%) | 15486 (0.03%) |
| red1-peel-2 | 53593148 | 51814834 (96.68%) | 255258 (0.48%) | 1508092 (2.81%) | 0 (0.00%) | 14964 (0.03%) |
| red1-peel-3 | 40000286 | 39449144 (98.62%) | 40066 (0.10%) | 511076 (1.28%) | 0 (0.00%) | 0 (0.00%) |
| red1-pulp-1 | 1.03E+08 | 100144792 (96.93%) | 201768 (0.20%) | 2958978 (2.86%) | 0 (0.00%) | 6412 (0.01%) |
| red1-pulp-2 | 1.05E+08 | 102787458 (97.61%) | 161814 (0.15%) | 2346790 (2.23%) | 0 (0.00%) | 6744 (0.01%) |
| red1-pulp-3 | 70068912 | 68958368 (98.42%) | 66808 (0.10%) | 1039284 (1.48%) | 0 (0.00%) | 4452 (0.01%) |
| red2-peel-1 | 51084712 | 50367994 (98.60%) | 51538 (0.10%) | 665180 (1.30%) | 0 (0.00%) | 0 (0.00%) |
| red2-peel-2 | 41120302 | 40676830 (98.92%) | 32132 (0.08%) | 411340 (1.00%) | 0 (0.00%) | 0 (0.00%) |
| red2-peel-3 | 42746728 | 42372448 (99.12%) | 34420 (0.08%) | 339860 (0.80%) | 0 (0.00%) | 0 (0.00%) |
| red2-pulp-1 | 42352044 | 42016192 (99.21%) | 72584 (0.17%) | 262940 (0.62%) | 0 (0.00%) | 328 (0.00%) |
| red2-pulp-2 | 52477912 | 52023532 (99.13%) | 95724 (0.18%) | 358202 (0.68%) | 0 (0.00%) | 454 (0.00%) |
| red2-pulp-3 | 59966572 | 59725034 (99.60%) | 59172 (0.10%) | 180384 (0.30%) | 0 (0.00%) | 1982 (0.00%) |
| red3-peel-1 | 72810110 | 72560124 (99.66%) | 50986 (0.07%) | 178798 (0.25%) | 0 (0.00%) | 20202 (0.03%) |
| red3-peel-2 | 58995040 | 58915280 (99.86%) | 34698 (0.06%) | 42502 (0.07%) | 0 (0.00%) | 2560 (0.00%) |
| red3-peel-3 | 56515710 | 56442620 (99.87%) | 31476 (0.06%) | 34170 (0.06%) | 0 (0.00%) | 7444 (0.01%) |
| red3-pulp-1 | 43641068 | 43305600 (99.23%) | 32186 (0.07%) | 303282 (0.69%) | 0 (0.00%) | 0 (0.00%) |
| red3-pulp-2 | 54784966 | 54414896 (99.32%) | 78384 (0.14%) | 291686 (0.53%) | 0 (0.00%) | 0 (0.00%) |
| red3-pulp-3 | 43332802 | 42952552 (99.12%) | 76526 (0.18%) | 303370 (0.70%) | 0 (0.00%) | 354 (0.00%) |
| red4-peel-1 | 44475390 | 44053894 (99.05%) | 32576 (0.07%) | 388920 (0.87%) | 0 (0.00%) | 0 (0.00%) |
| red4-peel-2 | 58665096 | 58459528 (99.65%) | 36118 (0.06%) | 152884 (0.26%) | 0 (0.00%) | 16566 (0.03%) |
| red4-peel-3 | 47215830 | 46603154 (98.70%) | 54178 (0.11%) | 558498 (1.18%) | 0 (0.00%) | 0 (0.00%) |
| red4-pulp-1 | 50384240 | 49794010 (98.83%) | 123272 (0.24%) | 466522 (0.93%) | 0 (0.00%) | 436 (0.00%) |
| red4-pulp-2 | 41431738 | 41142748 (99.30%) | 56376 (0.14%) | 232256 (0.56%) | 0 (0.00%) | 358 (0.00%) |
| red4-pulp-3 | 51372750 | 50893182 (99.07%) | 33690 (0.07%) | 445048 (0.87%) | 0 (0.00%) | 830 (0.00%) |
| white1-peel-1 | 43928854 | 43364900 (98.72%) | 46664 (0.11%) | 517290 (1.18%) | 0 (0.00%) | 0 (0.00%) |
| white1-peel-2 | 47768346 | 47213000 (98.84%) | 57912 (0.12%) | 496974 (1.04%) | 0 (0.00%) | 460 (0.00%) |
| white1-peel-3 | 47759042 | 47223172 (98.88%) | 58744 (0.12%) | 476664 (1.00%) | 0 (0.00%) | 462 (0.00%) |
| white1-pulp-1 | 43987280 | 43610962 (99.14%) | 77500 (0.18%) | 298426 (0.68%) | 0 (0.00%) | 392 (0.00%) |
| white1-pulp-2 | 59206148 | 58910828 (99.50%) | 73090 (0.12%) | 220236 (0.37%) | 0 (0.00%) | 1994 (0.00%) |
| white1-pulp-3 | 39939916 | 39267962 (98.32%) | 35342 (0.09%) | 636612 (1.59%) | 0 (0.00%) | 0 (0.00%) |
| white2-peel-1 | 60522262 | 60232118 (99.52%) | 44142 (0.07%) | 228686 (0.38%) | 0 (0.00%) | 17316 (0.03%) |
| white2-peel-2 | 41882728 | 41549266 (99.20%) | 30640 (0.07%) | 302822 (0.72%) | 0 (0.00%) | 0 (0.00%) |
| white2-peel-3 | 40638310 | 40278056 (99.11%) | 36630 (0.09%) | 323624 (0.80%) | 0 (0.00%) | 0 (0.00%) |
| white2-pulp-1 | 53251562 | 52801246 (99.15%) | 37968 (0.07%) | 411450 (0.77%) | 0 (0.00%) | 898 (0.00%) |
| white2-pulp-2 | 40309710 | 39948942 (99.11%) | 29144 (0.07%) | 331624 (0.82%) | 0 (0.00%) | 0 (0.00%) |
| white2-pulp-3 | 53923960 | 53471548 (99.16%) | 38804 (0.07%) | 412598 (0.77%) | 0 (0.00%) | 1010 (0.00%) |
| white3-peel-1 | 58709292 | 57725534 (98.32%) | 470720 (0.80%) | 496934 (0.85%) | 0 (0.00%) | 16104 (0.03%) |
| white3-peel-2 | 42246650 | 41815422 (98.98%) | 36504 (0.09%) | 394724 (0.93%) | 0 (0.00%) | 0 (0.00%) |
| white3-peel-3 | 54392976 | 53858890 (99.02%) | 43258 (0.08%) | 489892 (0.90%) | 0 (0.00%) | 936 (0.00%) |
| white3-pulp-1 | 42081538 | 41753230 (99.22%) | 25668 (0.06%) | 302640 (0.72%) | 0 (0.00%) | 0 (0.00%) |
| white3-pulp-2 | 49116476 | 48805708 (99.37%) | 98708 (0.20%) | 212060 (0.43%) | 0 (0.00%) | 0 (0.00%) |
| white3-pulp-3 | 46497804 | 46154772 (99.26%) | 81298 (0.17%) | 261364 (0.56%) | 0 (0.00%) | 370 (0.00%) |
| white4-peel-1 | 46789608 | 46262668 (98.87%) | 48054 (0.10%) | 478458 (1.02%) | 0 (0.00%) | 428 (0.00%) |
| white4-peel-2 | 47742188 | 47209434 (98.88%) | 45542 (0.10%) | 486734 (1.02%) | 0 (0.00%) | 478 (0.00%) |
| white4-peel-3 | 42502568 | 41975764 (98.76%) | 40826 (0.10%) | 485978 (1.14%) | 0 (0.00%) | 0 (0.00%) |
| white4-pulp-1 | 89792888 | 89268380 (99.42%) | 162718 (0.18%) | 361790 (0.40%) | 0 (0.00%) | 0 (0.00%) |
| white4-pulp-2 | 60550090 | 60077556 (99.22%) | 106610 (0.18%) | 365924 (0.60%) | 0 (0.00%) | 0 (0.00%) |
| white4-pulp-3 | 52887624 | 52287306 (98.86%) | 48936 (0.09%) | 550384 (1.04%) | 0 (0.00%) | 998 (0.00%) |

**Table S3.** Pathway enrichment analysis of important temporal profiles in peel and pulp of red and white pitaya fruits.

For red-peel

| B_Pathway | C_Pathway | profile1 | profile11 | profile15 | profile8 |
| --- | --- | --- | --- | --- | --- |
| Translation | ko03015 mRNA surveillance pathway | NA | 0.972975 | 0.934918 | 0.014084 |
| Lipid metabolism | ko00071 Fatty acid degradation | NA | 0.87505 | 0.008807 | 0.421076 |
| Signal transduction | ko04075 Plant hormone signal transduction | 9.28E-10 | NA | 0.881832 | 0.550918 |
| Global and overview maps | ko01100 Metabolic pathways | 2.84E-01 | 0.292239 | 4.96E-05 | 0.999678 |
| Amino acid metabolism | ko00280 Valine, leucine and isoleucine degradation | 8.89E-01 | 0.96022 | 4.96E-05 | 0.999678 |
| Biosynthesis of other secondary metabolites | ko00960 Tropane, piperidine and pyridine alkaloid biosynthesis | NA | NA | 0.008243 | 0.999678 |
| Metabolism of other amino acids | ko00480 Glutathione metabolism | NA | NA | 0.014274 | 0.999678 |
| Carbohydrate metabolism | ko00640 Propanoate metabolism | NA | NA | 0.016275 | 0.999678 |
| Metabolism of other amino acids | ko00460 Cyanoamino acid metabolism | 3.40E-02 | NA | 0.181474 | 0.999678 |
| Carbohydrate metabolism | ko00010 Glycolysis / Gluconeogenesis | 9.80E-01 | 0.035712 | 0.396859 | 0.999678 |
| Biosynthesis of other secondary metabolites | ko00940 Phenylpropanoid biosynthesis | 2.65E-02 | NA | 0.706108 | 0.999678 |
| Energy metabolism | ko00190 Oxidative phosphorylation | 7.91E-01 | 0.002271 | 0.736143 | 0.999678 |
| Energy metabolism | ko00710 Carbon fixation in photosynthetic organisms | 9.80E-01 | 0.012698 | 0.736143 | 0.999678 |
| Global and overview maps | ko01200 Carbon metabolism | 8.89E-01 | 0.035712 | 0.881832 | 0.999678 |
| Energy metabolism | ko00195 Photosynthesis | 6.26E-10 | NA | 0.881832 | 0.999678 |
| Energy metabolism | ko00196 Photosynthesis - antenna proteins | 7.88E-01 | 0.000866 | 0.883167 | 0.999678 |
| Carbohydrate metabolism | ko00030 Pentose phosphate pathway | 8.89E-01 | 0.010765 | 0.883167 | 0.999678 |
| Global and overview maps | ko01230 Biosynthesis of amino acids | 7.91E-01 | 0.012698 | 0.946169 | 0.999678 |
| Amino acid metabolism | ko00250 Alanine, aspartate and glutamate metabolism | 8.89E-01 | 0.019315 | 0.946169 | 0.999678 |
| Translation | ko03008 Ribosome biogenesis in eukaryotes | NA | 0.012698 | 0.992542 | 0.999678 |
| Translation | ko03010 Ribosome | NA | 0.002271 | 1 | 0.999678 |
| Biosynthesis of other secondary metabolites | ko00942 Anthocyanin biosynthesis | 3.40E-02 | NA | NA | 0.999678 |

For red pulp

| B_Pathway | C_Pathway | profile0 | profile12 | profile17 | profile18 | profile2 | profile7 |
| --- | --- | --- | --- | --- | --- | --- | --- |
| Biosynthesis of other secondary metabolites | ko00945 Stilbenoid, diarylheptanoid and gingerol biosynthesis | 4.22E-04 | NA | 9.56E-01 | NA | 3.20E-01 | 0.001622 |
| Global and overview maps | ko01110 Biosynthesis of secondary metabolites | 1.68E-10 | 0.710347 | 2.46E-02 | 0.996916 | 2.27E-04 | 0.001954 |
| Biosynthesis of other secondary metabolites | ko00940 Phenylpropanoid biosynthesis | 1.43E-13 | 0.564546 | 8.22E-01 | NA | 1.04E-03 | 0.004152 |
| Biosynthesis of other secondary metabolites | ko00941 Flavonoid biosynthesis | 2.37E-07 | NA | 8.22E-01 | NA | 5.08E-03 | 0.00547 |
| Signal transduction | ko04075 Plant hormone signal transduction | 2.12E-02 | 0.587336 | 2.06E-01 | 0.237529 | 5.04E-01 | 0.04038 |
| Global and overview maps | ko01100 Metabolic pathways | 9.51E-06 | 0.95331 | 1.01E-05 | 0.996916 | 4.89E-07 | 0.137074 |
| Biosynthesis of other secondary metabolites | ko00901 Indole alkaloid biosynthesis | 1.03E-03 | NA | NA | NA | 2.67E-01 | 0.177898 |
| Biosynthesis of other secondary metabolites | ko00965 Betalain biosynthesis | 3.52E-03 | NA | 2.55E-01 | NA | 3.03E-01 | 0.207848 |
| Biosynthesis of other secondary metabolites | ko00950 Isoquinoline alkaloid biosynthesis | 1.32E-03 | NA | 1.16E-01 | NA | 8.82E-02 | 0.252705 |
| Amino acid metabolism | ko00350 Tyrosine metabolism | 2.65E-03 | NA | 1.20E-01 | NA | 2.67E-01 | 0.432313 |
| Amino acid metabolism | ko00360 Phenylalanine metabolism | 8.63E-04 | NA | 2.16E-01 | NA | 3.03E-01 | 0.432313 |
| Environmental adaptation | ko04712 Circadian rhythm - plant | 7.72E-01 | NA | 4.37E-02 | 0.310943 | NA | 0.478366 |
| Carbohydrate metabolism | ko00040 Pentose and glucuronate interconversions | 5.43E-01 | 0.524849 | 3.97E-02 | NA | 1.92E-01 | 0.484522 |
| Lipid metabolism | ko00561 Glycerolipid metabolism | 3.91E-01 | NA | 9.79E-01 | 0.711117 | 2.55E-02 | 0.496042 |
| Lipid metabolism | ko00564 Glycerophospholipid metabolism | 7.58E-01 | NA | 9.56E-01 | 0.727316 | 5.44E-03 | 0.572964 |
| Carbohydrate metabolism | ko00500 Starch and sucrose metabolism | 3.53E-03 | NA | 8.15E-02 | 0.727316 | NA | 0.572964 |
| Lipid metabolism | ko00061 Fatty acid biosynthesis | NA | NA | 9.99E-01 | NA | 3.53E-03 | NA |
| Global and overview maps | ko01212 Fatty acid metabolism | 8.30E-01 | NA | 8.22E-01 | NA | 5.44E-03 | NA |
| Carbohydrate metabolism | ko00520 Amino sugar and nucleotide sugar metabolism | 9.03E-01 | 0.57196 | 8.22E-01 | 0.74207 | 4.31E-02 | NA |
| Metabolism of cofactors and vitamins | ko00130 Ubiquinone and other terpenoid-quinone biosynthesis | 1.80E-02 | NA | 9.56E-01 | NA | 2.67E-01 | NA |
| Metabolism of cofactors and vitamins | ko00860 Porphyrin and chlorophyll metabolism | NA | NA | 4.37E-02 | NA | 6.52E-01 | NA |
| Metabolism of other amino acids | ko00410 beta-Alanine metabolism | 7.58E-01 | NA | 3.97E-02 | NA | NA | NA |

For white peel

| B_Pathway | C_Pathway | profile0 | profile1 | profile17 | profile2 | profile3 | profile5 | profile7 |
| --- | --- | --- | --- | --- | --- | --- | --- | --- |
| Signal transduction | ko04075 Plant hormone signal transduction | 0.024112 | 5.97E-01 | 0.320212 | 1.76E-01 | NA | 0.688101 | 0.030439 |
| Global and overview maps | ko01110 Biosynthesis of secondary metabolites | 0.120367 | 3.75E-06 | 0.215122 | 7.60E-02 | 0.016634 | 0.708308 | 0.030439 |
| Signal transduction | ko04016 MAPK signaling pathway - plant | 0.588252 | 8.55E-01 | 0.540919 | 7.67E-01 | 0.811228 | NA | 0.030439 |
| Global and overview maps | ko01230 Biosynthesis of amino acids | 0.504507 | 2.63E-01 | 0.890553 | 5.52E-01 | 0.307764 | 0.688101 | 0.042172 |
| Global and overview maps | ko01100 Metabolic pathways | 0.268509 | 3.58E-04 | 0.320212 | 7.60E-02 | 0.040764 | 0.776636 | 0.188835 |
| Carbohydrate metabolism | ko00052 Galactose metabolism | 0.013502 | 1.52E-01 | 0.540919 | 9.64E-01 | 0.307764 | NA | 0.288852 |
| Amino acid metabolism | ko00260 Glycine, serine and threonine metabolism | NA | 2.42E-02 | 0.914351 | 7.12E-01 | NA | NA | 0.289216 |
| Carbohydrate metabolism | ko00030 Pentose phosphate pathway | NA | 9.93E-03 | NA | 1.00E+00 | 0.452888 | 0.708308 | 0.676113 |
| Biosynthesis of other secondary metabolites | ko00940 Phenylpropanoid biosynthesis | 0.557938 | 9.48E-02 | 0.320212 | 7.48E-01 | 0.00442 | 0.786404 | 0.862726 |
| Energy metabolism | ko00195 Photosynthesis | NA | NA | 0.914351 | 1.62E-07 | NA | 0.369999 | NA |
| Biosynthesis of other secondary metabolites | ko00941 Flavonoid biosynthesis | NA | 5.97E-01 | 0.540919 | 8.13E-01 | 0.016634 | NA | NA |
| Biosynthesis of other secondary metabolites | ko00945 Stilbenoid, diarylheptanoid and gingerol biosynthesis | NA | 6.17E-01 | 0.625426 | 8.13E-01 | 0.016634 | NA | NA |
| Biosynthesis of other secondary metabolites | ko00942 Anthocyanin biosynthesis | NA | 4.79E-02 | NA | 4.00E-01 | NA | NA | NA |

For white pulp

| B_Pathway | C_Pathway | profile0 | profile12 | profile17 | profile18 | profile2 | profile4 | profile5 |
| --- | --- | --- | --- | --- | --- | --- | --- | --- |
| Translation | ko03010 Ribosome | NA | 0.924675 | NA | 0.999886 | 0.999991 | 9.95E-01 | 0.028532 |
| Global and overview maps | ko01110 Biosynthesis of secondary metabolites | 0.005123 | 0.897711 | 0.207866 | 0.797369 | 7.61E-05 | 5.45E-06 | 0.860725 |
| Global and overview maps | ko01100 Metabolic pathways | 0.146096 | 0.924675 | 0.553745 | 0.797369 | 0.00118 | 3.20E-04 | 0.860725 |
| Metabolism of other amino acids | ko00480 Glutathione metabolism | NA | NA | 0.715433 | 0.754658 | 0.951077 | 2.47E-03 | 0.860725 |
| Global and overview maps | ko01212 Fatty acid metabolism | 0.684764 | 0.867246 | NA | 0.893114 | 0.05459 | 3.49E-03 | 0.860725 |
| Global and overview maps | ko01200 Carbon metabolism | 0.858658 | 0.867246 | 0.756246 | 0.786331 | 0.951077 | 3.49E-03 | 0.860725 |
| Lipid metabolism | ko00061 Fatty acid biosynthesis | NA | 0.867246 | NA | 0.862697 | 0.174893 | 3.11E-02 | 0.860725 |
| Biosynthesis of other secondary metabolites | ko00940 Phenylpropanoid biosynthesis | 0.00025 | NA | 0.546775 | 0.786331 | 0.000335 | 8.06E-02 | 0.860725 |
| Carbohydrate metabolism | ko00052 Galactose metabolism | 0.392084 | 0.032143 | NA | NA | 0.147728 | 3.95E-01 | 0.860725 |
| Lipid metabolism | ko01040 Biosynthesis of unsaturated fatty acids | NA | 0.867246 | NA | NA | 0.002145 | 6.75E-01 | 0.860725 |
| Transcription | ko03040 Spliceosome | NA | 0.005852 | 0.921679 | 0.935762 | NA | NA | 0.860725 |
| Signal transduction | ko04075 Plant hormone signal transduction | 0.049312 | 0.867246 | 0.032502 | 0.836907 | 0.008414 | 2.08E-01 | 0.882086 |
| Global and overview maps | ko01230 Biosynthesis of amino acids | 0.802859 | 0.867246 | 0.715433 | 0.786331 | 0.951077 | 5.79E-03 | 0.902625 |
| Biosynthesis of other secondary metabolites | ko00942 Anthocyanin biosynthesis | 0.159098 | NA | 0.032161 | NA | NA | NA | NA |

**Table S4.** Expression trend analysis of key genes in betalain biosynthesis pathways. Only gene expressed by red pulp were group into significant profiles which are profile0.

| Group | ko_ID | gene_ID | annotation | trend_peel | peel_1 | peel_2 | peel_3 | peel_4 | trend_pulp | pulp_1 | pulp_2 | pulp_3 | pulp_4 |
| --- | --- | --- | --- | --- | --- | --- | --- | --- | --- | --- | --- | --- | --- |
| Red | K01593 | Unigene0016763 | DDC, TDC; aromatic-L-amino-acid/L-tryptophan decarboxylase [EC:4.1.1.28 4.1.1.105] | N/A | N/A | N/A | N/A | N/A | 2 | 0 | -8.58 | -8.58 | -8.58 |
| Red | K01593 | Unigene0038605 | DDC, TDC; aromatic-L-amino-acid/L-tryptophan decarboxylase [EC:4.1.1.28 4.1.1.105] | 1 | 0 | -5.11 | -7.87 | -3.98 | 0 | 0 | -5.76 | -6.65 | -13.59 |
| Red | K01593 | Unigene0038606 | DDC, TDC; aromatic-L-amino-acid/L-tryptophan decarboxylase [EC:4.1.1.28 4.1.1.105] | 1 | 0 | -5.89 | -6.98 | -3.95 | 0 | 0 | -6.08 | -6.79 | -12.86 |
| Red | K01593 | Unigene0060913 | DDC, TDC; aromatic-L-amino-acid/L-tryptophan decarboxylase [EC:4.1.1.28 4.1.1.105] | N/A | N/A | N/A | N/A | N/A | 7 | 0 | 0 | -3.75 | -5.54 |
| Red | K01593 | Unigene0076738 | DDC, TDC; aromatic-L-amino-acid/L-tryptophan decarboxylase [EC:4.1.1.28 4.1.1.105] | N/A | N/A | N/A | N/A | N/A | 0 | 0 | -1.05 | -1.32 | -1.94 |
| Red | K15777 | Unigene0053908 | DOPA; 4,5-DOPA dioxygenase extradiol [EC:1.13.11.-] | 15 | 0 | 3.24 | -1.44 | 2.91 | 17 | 0 | 3.35 | 3.6 | 2.91 |
| Red | K15777 | Unigene0068354 | DOPA; 4,5-DOPA dioxygenase extradiol [EC:1.13.11.-] | 15 | 0 | 4.72 | 0.6 | 4.98 | 17 | 0 | 4.75 | 4.2 | 3.17 |
| Red | K15777 | Unigene0068355 | DOPA; 4,5-DOPA dioxygenase extradiol [EC:1.13.11.-] | 15 | 0 | 5.27 | 1.26 | 5.68 | 17 | 0 | 5.4 | 4.5 | 3.33 |
| White | K01593 | Unigene0001945 | DDC, TDC; aromatic-L-amino-acid/L-tryptophan decarboxylase [EC:4.1.1.28 4.1.1.105] | 1 | 0 | -7.23 | -8.11 | -3.89 | 2 | 0 | -8.46 | -7.53 | -8.23 |
| White | K01593 | Unigene0028665 | DDC, TDC; aromatic-L-amino-acid/L-tryptophan decarboxylase [EC:4.1.1.28 4.1.1.105] | 6 | 0 | -6.69 | -1.93 | 3.25 | N/A | N/A | N/A | N/A | N/A |
| White | K01593 | Unigene0028666 | DDC, TDC; aromatic-L-amino-acid/L-tryptophan decarboxylase [EC:4.1.1.28 4.1.1.105] | 8 | 0 | 1.32 | -6.69 | 3.42 | N/A | N/A | N/A | N/A | N/A |
| White | K01593 | Unigene0034824 | DDC, TDC; aromatic-L-amino-acid/L-tryptophan decarboxylase [EC:4.1.1.28 4.1.1.105] | 5 | 0 | -3.61 | -1.92 | -1.36 | 2 | 0 | -2.61 | -1.93 | -2.08 |
| White | K01593 | Unigene0068289 | DDC, TDC; aromatic-L-amino-acid/L-tryptophan decarboxylase [EC:4.1.1.28 4.1.1.105] | 10 | 0 | 0.43 | 0.3 | 2.17 | N/A | N/A | N/A | N/A | N/A |
| White | K15777 | Unigene0004044 | DOPA; 4,5-DOPA dioxygenase extradiol [EC:1.13.11.-] | 17 | 0 | 2.95 | 1.54 | 1.52 | N/A | N/A | N/A | N/A | N/A |
| White | K15777 | Unigene0026204 | DOPA; 4,5-DOPA dioxygenase extradiol [EC:1.13.11.-] | 14 | 0 | 1.31 | 0.34 | 0.54 | N/A | N/A | N/A | N/A | N/A |
