# Supplementary material for: Comparative transcriptomic profiling in the pulp and peel of pitaya fruit uncovers the gene networks regulating pulp color formation
Source: Front Plant Sci. 2022 Aug 3;13:968925. doi: 10.3389/fpls.2022.968925 (PMC9382024; doi:10.3389/fpls.2022.968925)
Supplement: Supplementary Figure 2 — The specific top 20 GO terms for each comparison. [file Data_Sheet_2.PDF]

DAP20 Vs DAP25 | DAP25 Vs DAP30 | DAP30 Vs DAP35

Red Peel

ID	Descrption	ID	Descrption	ID	Descrption
GO:0042221	response to chemical	GO:0009451	RNA modification	GO:0009451	RNA modification
GO:0050896	response to stimulus	GO:0022613	ribonucleoprotein complex biogenesis	GO:0022613	ribonucleoprotein complex biogenesis
GO:0055082	cellular chemical homeostasis	GO:0010467	gene expression	GO:0044281	small molecule metabolic process
GO:0006873	cellular ion homeostasis	GO:0009117	nucleotide metabolic process	GO:0044391	ribosomal subunit
GO:0009891	positive regulation of biosynthetic process	GO:0030529	intracellular ribonucleoprotein complex	GO:0005840	ribosome
GO:0034285	response to disaccharide	GO:1990904	ribonucleoprotein complex	GO:0030529	intracellular ribonucleoprotein complex
GO:0065007	biological regulation	GO:0005840	ribosome	GO:1990904	ribonucleoprotein complex
GO:0050801	ion homeostasis	GO:0044391	ribosomal subunit	GO:0015934	large ribosomal subunit
GO:0009416	response to light stimulus	GO:0015934	large ribosomal subunit	GO:0043228	non−membrane−bounded organelle
GO:0048878	chemical homeostasis	GO:0032991	macromolecular complex	GO:0043232	intracellular non−membrane−bounded organelle
GO:0044699	single−organism process	GO:0043228	non−membrane−bounded organelle	GO:0032991	macromolecular complex
GO:0010033	response to organic substance	GO:0043232	intracellular non−membrane−bounded organelle	GO:0005737	cytoplasm
GO:1901700	response to oxygen−containing compound	GO:0005737	cytoplasm	GO:0044444	cytoplasmic part
GO:0009314	response to radiation	GO:0044444	cytoplasmic part	GO:0044422	organelle part
GO:0019725	cellular homeostasis	GO:0044422	organelle part	GO:0044446	intracellular organelle part
GO:0009507	chloroplast	GO:0031090	organelle membrane	GO:0031090	organelle membrane
GO:0044434	chloroplast part	GO:0005911	cell−cell junction	GO:0015935	small ribosomal subunit
GO:0044435	plastid part	GO:0030054	cell junction	GO:0005911	cell−cell junction
GO:0009579	thylakoid	GO:0015935	small ribosomal subunit	GO:0030054	cell junction
GO:0016491	oxidoreductase activity	GO:0005198	structural molecule activity	GO:0005198	structural molecule activity

Red Pulpl

ID	Descrption	ID	Descrption	ID	Descrption
GO:0042221	response to chemical	GO:0009719	response to endogenous stimulus	GO:0009719	response to endogenous stimulus
GO:0050896	response to stimulus	GO:0010243	response to organonitrogen compound	GO:0009692	ethylene metabolic process
GO:0001101	response to acid chemical	GO:0010033	response to organic substance	GO:0043449	cellular alkene metabolic process
GO:0009698	phenylpropanoid metabolic process	GO:0042221	response to chemical	GO:1900673	olefin metabolic process
GO:0009699	phenylpropanoid biosynthetic process	GO:0050896	response to stimulus	GO:0010243	response to organonitrogen compound
GO:0006950	response to stress	GO:0009642	response to light intensity	GO:0065007	biological regulation
GO:0044550	secondary metabolite biosynthetic process	GO:0009620	response to fungus	GO:0009725	response to hormone
GO:0009891	positive regulation of biosynthetic process	GO:1901698	response to nitrogen compound	GO:0002252	immune effector process
GO:1901700	response to oxygen−containing compound	GO:0009725	response to hormone	GO:0009685	gibberellin metabolic process
GO:0019748	secondary metabolic process	GO:0001101	response to acid chemical	GO:0051716	cellular response to stimulus
GO:0044699	single−organism process	GO:1901700	response to oxygen−containing compound	GO:0010033	response to organic substance
GO:0009812	flavonoid metabolic process	GO:0006950	response to stress	GO:0016101	diterpenoid metabolic process
GO:0009813	flavonoid biosynthetic process	GO:0000302	response to reactive oxygen species	GO:1901698	response to nitrogen compound
GO:0009628	response to abiotic stimulus	GO:0035556	intracellular signal transduction	GO:0000103	sulfate assimilation
GO:0009605	response to external stimulus	GO:0006979	response to oxidative stress	GO:0018871	1−aminocyclopropane−1−carboxylate metabolic process
GO:0006595	polyamine metabolic process	GO:0032870	cellular response to hormone stimulus	GO:0019755	one−carbon compound transport
GO:0009416	response to light stimulus	GO:0071495	cellular response to endogenous stimulus	GO:0070542	response to fatty acid
GO:0065007	biological regulation	GO:0009755	hormone−mediated signaling pathway	GO:0007165	signal transduction
GO:0051716	cellular response to stimulus	GO:0043207	response to external biotic stimulus	GO:0023052	signaling
GO:0016491	oxidoreductase activity	GO:0005618	cell wall	GO:0044700	single organism signaling

White Peel

ID	Descrption	ID	Descrption	ID	Descrption
GO:0042221	response to chemical	GO:0009719	response to endogenous stimulus	GO:0006950	response to stress
GO:0044707	single−multicellular organism process	GO:0009725	response to hormone	GO:1901700	response to oxygen−containing compound
GO:0032501	multicellular organismal process	GO:0009755	hormone−mediated signaling pathway	GO:0042221	response to chemical
GO:1901700	response to oxygen−containing compound	GO:0032870	cellular response to hormone stimulus	GO:0051704	multi−organism process
GO:0044283	small molecule biosynthetic process	GO:0071495	cellular response to endogenous stimulus	GO:0050896	response to stimulus
GO:0007275	multicellular organism development	GO:0001101	response to acid chemical	GO:0009642	response to light intensity
GO:0048856	anatomical structure development	GO:0009827	plant−type cell wall modification	GO:0009607	response to biotic stimulus
GO:0044767	single−organism developmental process	GO:0015833	peptide transport	GO:0051707	response to other organism
GO:0010033	response to organic substance	GO:0042886	amide transport	GO:0044550	secondary metabolite biosynthetic process
GO:0050896	response to stimulus	GO:0044283	small molecule biosynthetic process	GO:0000302	response to reactive oxygen species
GO:0006790	sulfur compound metabolic process	GO:0010033	response to organic substance	GO:0043207	response to external biotic stimulus
GO:0044699	single−organism process	GO:0001071	nucleic acid binding transcription factor activity	GO:0009814	defense response, incompatible interaction
GO:0044711	single−organism biosynthetic process	GO:0003677	DNA binding	GO:0019748	secondary metabolic process
GO:0009416	response to light stimulus	GO:0015144	carbohydrate transmembrane transporter activity	GO:0009605	response to external stimulus
GO:0015979	photosynthesis	GO:1901476	carbohydrate transporter activity	GO:0098542	defense response to other organism
GO:0005618	cell wall	GO:0019842	vitamin binding	GO:0009699	phenylpropanoid biosynthetic process
GO:0030312	external encapsulating structure	GO:0016628	oxidoreductase activity, acting on the CH−CH group of donors, NAD or NADP as acceptor	GO:0006979	response to oxidative stress
GO:0071944	cell periphery	GO:0016641	oxidoreductase activity, acting on the CH−NH2 group of donors, oxygen as acceptor	GO:0009698	phenylpropanoid metabolic process
GO:0005576	extracellular region	GO:0000975	regulatory region DNA binding	GO:0030312	external encapsulating structure
GO:0016491	oxidoreductase activity	GO:0001067	regulatory region nucleic acid binding	GO:0071944	cell periphery

White Pulp

ID	Descrption	ID	Descrption	ID	Descrption
GO:0050896	response to stimulus	GO:0000314	organellar small ribosomal subunit	GO:0000302	response to reactive oxygen species
GO:0042221	response to chemical	GO:0044391	ribosomal subunit	GO:0006979	response to oxidative stress
GO:0006950	response to stress	GO:0015935	small ribosomal subunit	GO:0009642	response to light intensity
GO:0009628	response to abiotic stimulus	GO:0000313	organellar ribosome	GO:0042221	response to chemical
GO:0001101	response to acid chemical	GO:0000152	nuclear ubiquitin ligase complex	GO:0050896	response to stimulus
GO:1901700	response to oxygen−containing compound	GO:0005840	ribosome	GO:1901700	response to oxygen−containing compound
GO:0006575	cellular modified amino acid metabolic process	GO:0016021	integral component of membrane	GO:0009416	response to light stimulus
GO:0006595	polyamine metabolic process	GO:0030529	intracellular ribonucleoprotein complex	GO:0009314	response to radiation
GO:0044106	cellular amine metabolic process	GO:1990904	ribonucleoprotein complex	GO:0009408	response to heat
GO:0065007	biological regulation	GO:0003723	RNA binding	GO:0006950	response to stress
GO:0006576	cellular biogenic amine metabolic process	GO:0070566	adenylyltransferase activity	GO:0009628	response to abiotic stimulus
GO:0009698	phenylpropanoid metabolic process	GO:0003676	nucleic acid binding	GO:0009266	response to temperature stimulus
GO:0010033	response to organic substance	GO:0015930	glutamate synthase activity	GO:0010029	regulation of seed germination
GO:0009719	response to endogenous stimulus	GO:0045181	glutamate synthase activity, NAD(P)H as acceptor	GO:1900140	regulation of seedling development
GO:0000302	response to reactive oxygen species	GO:0052736	beta−glucanase activity	GO:0033554	cellular response to stress
GO:0009416	response to light stimulus	GO:0044822	poly(A) RNA binding	GO:0051716	cellular response to stimulus
GO:0009314	response to radiation	GO:0019842	vitamin binding	GO:0009845	seed germination
GO:0044699	single−organism process	GO:0004779	sulfate adenylyltransferase activity	GO:0030312	external encapsulating structure
GO:0009642	response to light intensity	GO:0005198	structural molecule activity	GO:0005576	extracellular region
GO:0016491	oxidoreductase activity	GO:0015103	inorganic anion transmembrane transporter activity	GO:0071944	cell periphery
