# Supplementary material for: Comparative transcriptomic profiling in the pulp and peel of pitaya fruit uncovers the gene networks regulating pulp color formation
Source: Front Plant Sci. 2022 Aug 3;13:968925. doi: 10.3389/fpls.2022.968925 (PMC9382024; doi:10.3389/fpls.2022.968925)
Supplement: Supplementary Figure 4 — KEGG pathway enrichment analysis of DE genes of white fruits during growth. [file Data_Sheet_4.PDF]

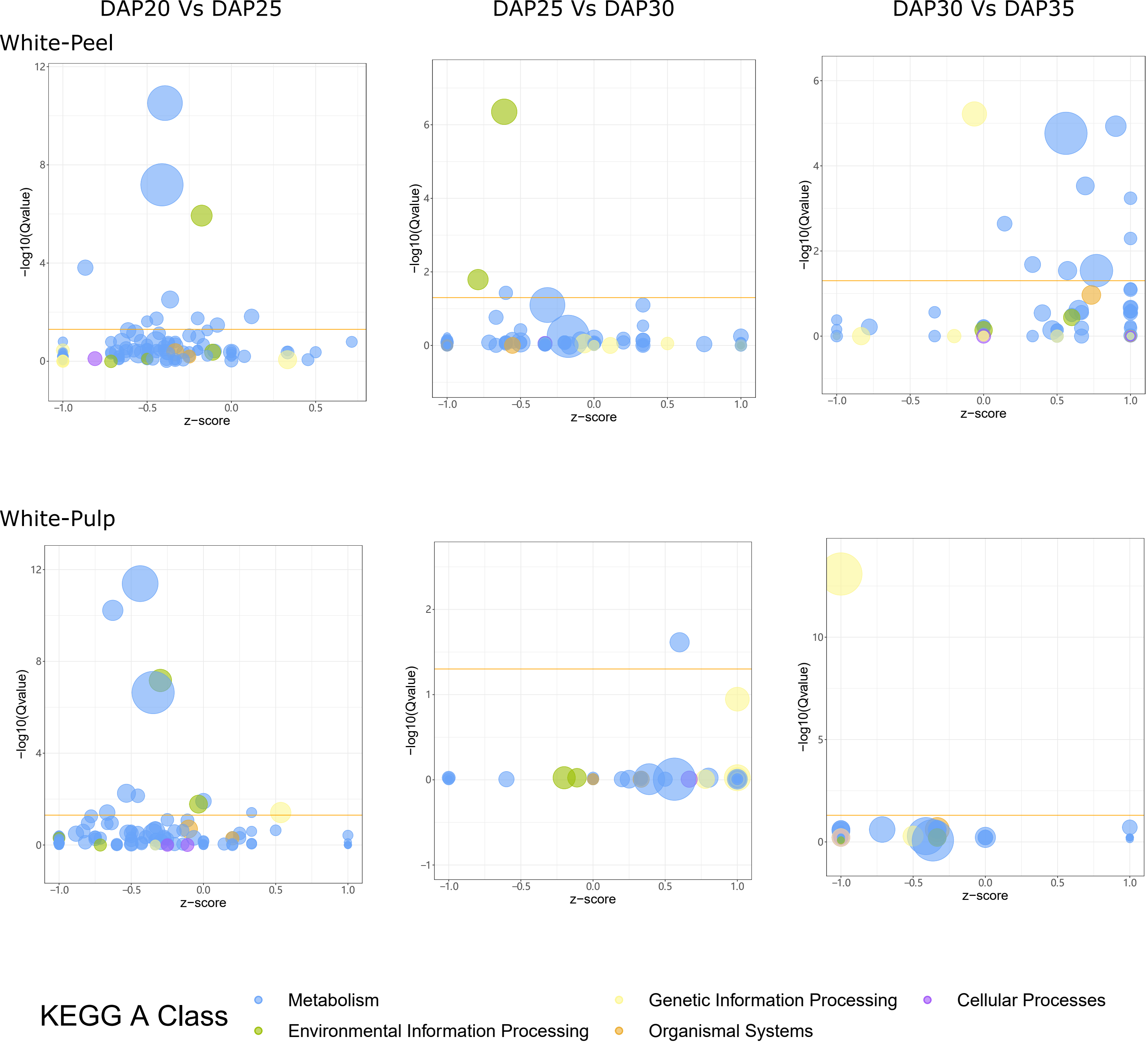

DAP20 Vs DAP25
DAP25 Vs DAP30
DAP30 Vs DAP35
White-Peel
White-Pulp
−log10(Qvalue)
z−score
KEGG A Class
Metabolism
Genetic Information Processing
Cellular Processes
Environmental Information Processing
Organismal Systems
